# Quantitative Evaluation of Tactile Foraging Behavior in Pekin and Muscovy Ducks

**DOI:** 10.3389/fphys.2022.921657

**Published:** 2022-06-14

**Authors:** Aaron K. West, Emily M. Xu, Mitchell D. Nelson, Thomas R. Hart, Emelia M. Stricker, Alexandra G. Cones, Grace M. Martin, Kourtney Strickland, Devin L. Lambert, Lainey Burman, Bailey H. Zhu, Eve R. Schneider

**Affiliations:** Biology Department, University of Kentucky, Lexington, KY, United States

**Keywords:** Anseriformes, foraging behavior, *Cairina moschata*, tactile, duck bill, *Anas platyrhnchos*, touch

## Abstract

Ducks have developed a variety of foraging strategies that utilize touch sensitive bills to match their ecological niche within wetlands. These techniques include diving, sieving, dabbling, and grazing. Ducks exhibiting tactile specialization in foraging outperform visual and non-tactile foraging ducks in behavioral experiments and have a higher percentage of light-touch mechanoreceptor neurons expressing Piezo2 in the trigeminal ganglia. Belonging to two different tribes of Anseriformes, the well-studied tactile specialist Pekin (Tribe Anatini: *Anas platyrhynchos domestica*) and lesser studied Muscovy (Tribe Cairinini: *Cairina moschata domestica*) ducks were tested on a series of experiments to assess these birds’ functional tactile acuity. Both species of duck were able to separate out and consume edible items from increasing amounts of inedible plastiline clay distractors. They could also both be trained to associate a food reward with plastiline stimuli of differing size and shape using touch alone. However, only females of each species could learn to associate food reward with otherwise identical stimuli differing only in hardness. Pekin females performed significantly better than Muscovy females suggesting the anatomical specializations present in many Anatini may contribute to this type of tactile acuity. These findings have potential relevance in understanding the evolution of tactile ability and feeding ecology.

## 1 Introduction

Ducks are a group within the family Anatidae that display a diversity of specializations in feeding. The diets of these birds include an array of both plant matter (acorns, seeds, grasses, roots, leaves, etc.) and animal prey (mollusks, aquatic insects, eggs, fish, crabs, plankton, etc.) (Baldassarre et al., 2014). Ducks have developed a variety of foraging strategies to match their ecological niche. Several ducks dive for food, such as the lesser scaup (*Aythya affinis*) and ruddy duck (*Oxyura jamaicensis*) (Tome and Wrubleski, 1988), which sieve through underwater sediment, often in turbid conditions, in search of aquatic invertebrates. Others, such as the mallard, feed by straining for food at the surface or dipping their heads into the water to forage (behaviors known as “dabbling”). The technique of straining food items from water or mud is common in most ducks and is aided in part by specialized mouth morphology. Analysis of beak curvature in waterfowl shows a strong correlation between beak morphology and feeding ecology, with wider “duck-like” beaks being associated with filter feeding (Olsen, 2017). Foraging by ducks is commonly performed in conditions of poor visibility, suggesting that tactile acuity in addition to beak morphology is critical for foraging (Mcneil et al., 1992). Recently, we examined anatomical and molecular signatures of tactile foraging ability across a range of species reported to have different foraging strategies. Trigeminal ganglia, which contain mechanosensitive neurons that innervate the bill, contain higher percentages of cells expressing the ion channel Piezo2 in tactile foraging species compared to species that use visual or non-specialized foraging methods (Schneider et al., 2014; Schneider et al., 2017). The mallard duck (*A. platyrhynchos*) and its domesticated descendent the Pekin duck (*A. platyrhynchos domestica*) are considered champion tactile foragers, with similarly high numbers of Piezo2-positive neurons in the trigeminal ganglia. Additionally, in a study examining late-stage duck embryos of seven species, the domestic duck had the fewest number of neurons expressing molecular markers consistent with a thermoreceptor/nociceptor phenotype, suggesting an evolutionary tradeoff between sensing light-touch and temperature (Schneider et al., 2019). Behaviorally, mallards are adept tactile foragers, capable of harvesting peas in wet sand while avoiding similar size inedible distractors (plastiline balls) (Zweers et al., 1977). They outperform non-tactile foraging wigeons (*Mareca penelope*) and visually foraging white-fronted geese (*Anser albifrons*) in filter-feeding tasks (Van Der Leeuw et al., 2003). Another study demonstrated that anesthetizing the bill significantly increases the time it takes for mallards to catch a tadpole in darkness (Avilova, 2017). However, most duck species dabble as ducklings regardless of their specialization in adulthood (Collias, 1958), and many grazing species dabble for insect larvae during breeding season (Drobney and Fredrickson, 1979). Thus, we wondered how closely linked tactile acuity is to adaptations in the anatomy of the trigeminal system.

To evaluate this, it is first necessary to develop behavioral tests disentangling ability from preference or other environmental factors. As our comparison species, we chose two domesticated ducks belonging to two distantly-related tribes: the well-known Pekin duck and the Muscovy duck (*C. moschata domestica*)— the only domestic duck not descended from mallard (Donkin, 1989), both of which have fully sequenced genomes. Muscovy ducks are members of the tribe Cairinini (perching ducks). Others in this tribe, such as the wood duck (*Aix sponsa*) and mandarin duck (*Aix galliculata*) display anatomical features consistent with a visual rather than tactile foraging strategy. For instance, wood ducks have large eyes, hooked bills, and small ratios of PrV (principal sensory nucleus of the trigeminal nerve) to the size of visual brain regions such as nucleus rotundus, or whole brain (Dubbeldam, 1990; Gutierrez-Ibanez et al., 2009). In a study by Olsen (2017) in which three-dimensional beak curvature was measured and compared across 43 waterfowl species, Muscovy ducks were shown to have a slightly more “goose-like” beak in comparison to the wide “duck-like” bill of mallards (Olsen, 2017). Muscovy ducks are considered generalists, often foraging by grazing. There are few studies of foraging ecology in the Muscovy duck and none of these studies directly assess tactile foraging ability (Baldassarre et al., 2014). Thus, we chose to compare Pekin and Muscovy ducks on a battery of behavioral tasks to assess tactile ability. Ducks were trained to forage in muddy water to eliminate the use of visual cues and assess their abilities to discriminate submerged food items from non-food items. Trials in clear water were used to assess possible reliance on visual cues by the species. To rule out the possibility of taste or olfactory cues playing a role in behavioral performance, two-alternative choice tasks were designed in which ducks were trained to associate food rewards with objects of different shape, size, and hardness. We predicted that in the tactile tasks, but not visual tasks, Pekin ducks would outperform Muscovy ducks.

## 2 Results

**FIGURE 1:**
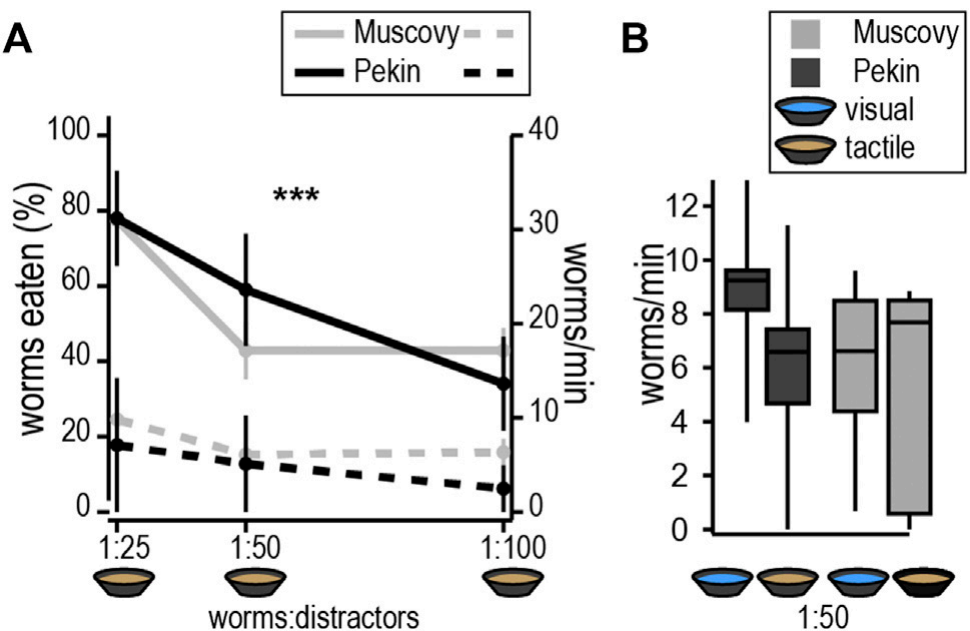
Increasing difficulty of tactile foraging tasks. **(A)** Performance of target consumption (mealworms eaten, solid line, left y-axis) and non-target consumption (distractors eaten, dashed line, right y-axis) throughout tasks of increasing difficulty. Ratios of worms:distractors are calculated from weights in grams used in each task. Both Pekins and Muscovies consumed fewer worms as the ratio of distractors increased [F(2,39) = 8.5, *p* < 0.0001]. **(B)** Foraging-time corrected measurements (worms/min) showed no change as a function of the number of distractors. Conditions that allow ducks to see the worms did not improve task performance in either species.

Experiments were conducted to assess ducks’ ability to pick out mealworms from among inedible distractors of a similar texture but slightly smaller diameter at various ratios of mealworms to distractors (Figure 1A). Both species ate less than 2% of the plastiline across all conditions (Muscovy = 0.78 ± 0.23 pieces, Pekin = 1.07 ± 0.55 pieces, n. s.). Contrary to our predictions, there was no significant difference between species in either the rate of consuming mealworms or the percentage of mealworms eaten [F(2,24) = 3.94, F(1,22) = 0.005, n. s.]. There was, however, a significant random effect of subjects in number, but not rate, of worms eaten (Wald test, *p* < 0.05), but this did not persist when corrected for the time spent foraging. As the ratio of distractors to mealworms increased, Pekins and Muscovies both consumed fewer worms [F(2,39) = 8.5, *p* < 0.0001, Figure 1A]. The only interspecies difference we observed was that Muscovies foraged for significantly less time than Pekins across all conditions [F(1,27) = 8.74, *p* < 0.01]. These data demonstrate that like the Pekin, Muscovy can separate edible items (worms) from inedible distractors of similar size and shape.

We then asked whether foraging performance of Muscovies would improve, compared to Pekins, under conditions that allowed ducks to use both touch and vision (clear water) (Figure 1B). Surprisingly, the number of worms eaten per minute was not significantly different between clear and muddy water for either species (Species x condition, F(1,21) = 0.35, *p* = *n*. s.), suggesting that adding vision did not improve performance.

**FIGURE 2:**
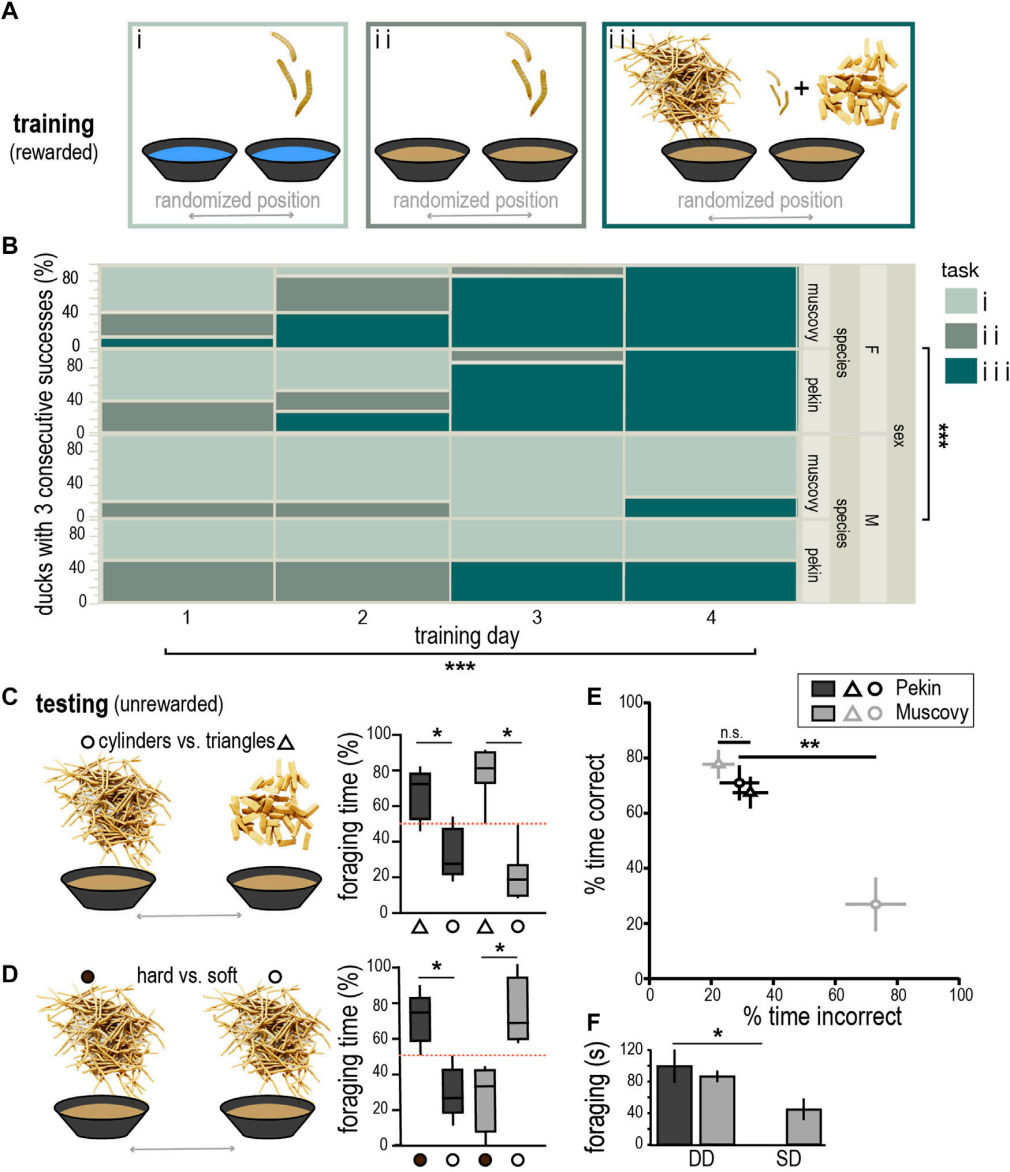
Training and performance on tactile conditioning tasks. **(A)** Schematic of progressively more difficult tasks (i–iii) used to train ducks to learn shape discrimination (images of worms and plastiline not to scale with bowls, worms in i and ii = 2x scale from iii). **(B)** Progression of training on tasks i-iii indicating majority of ducks learned task iii after four training days [nominal logistic fit, Chi^2^ (sex) = 16.7, df = 2; Chi^2^ (training day) = 23.8, df = 6]. **(C)** Schematic of testing condition and box and whisker plot showing % foraging time in the correct bowl (triangle symbol) or incorrect bowl (circle), red dashed line indicates chance performance, whiskers indicate 10–90 percentile. **(D)** Schematic of hard (filled circles) vs. soft (open circles) testing condition, and box and whisker plot showing behavioral performance as in **(C)**. **(E)** Cross-species comparison of % time spent in the correct (hard) vs. incorrect (soft) bowls for Pekin (black) and Muscovy (grey). **(F)** Pekin ducks spent significantly more time doing deep dabbling (DD) than surface dabbling/gleaning (SD) on this task, whereas the Muscovy used both foraging strategies. Asterisk indicate *p < 0.5, **p < 0.01, ***p < 0.0001.

To rule out taste and olfactory cues, ducks were trained to associate food reward with plastiline distractors in a two-choice foraging task (Figure 2A). Interestingly, significantly more females than males were able to learn this task after 3–4 days of training (Six females and one male for each species, nominal logistic fit, Chi^2^ = 16.7, *p* < 0.0001, Figure 2B). Both species spent significantly more time exploring the shape associated with reward (triangles) than the unrewarded shape (cylinders), demonstrating they had learned to associate large plastiline triangles with reward (Pekin: *t* = 4.29, df = 10, *p* < 0.05, Muscovy: *t* = 7.56, df = 12, *p* < 0.001, Figure 2C). The fact they could make this association demonstrates that ducks could differentiate between objects of differing size and shape using touch. Additionally, there was no significant difference in the percentage of time the two species spent foraging in the correct bowl (*t* = 1.3, df = 11, *p* = 0.21). However, when restricting our analysis to the first minute of the task, Muscovy ducks performed significantly better than Pekin (*t* = 2.7, df = 11, *p* < 0.05).

Ducks successful on the previous task were tested on whether they could associate a food reward with objects differing only in hardness. Consistent with our predictions, only Pekins could successfully do the task on the unrewarded test trial, spending an average of 76% of the time foraging in the correct bowl (*t* = 4.6, df = 8, *p* < 0.005, Figure 2D), whereas Muscovies spent only an average of 27% of the time foraging in the correct bowl and spent significantly more time foraging in the incorrect bowl (*t* = 3.3, df = 6, *p* < 0.05). Thus, Pekins spent significantly more time in the correct bowl than Muscovies (*t* = 3.9, df = 7, *p* < 0.01, Figure 2E). This result suggests Pekin ducks have superior tactile discrimination ability that can be revealed by tasks of sufficient difficulty.

In addition to differing performance on this task, a closer examination revealed a significant difference in foraging behavior between the species (Kruskal-Wallis test, *p* < 0.001). Post-hoc comparison revealed that Pekins almost exclusively used a “deep dabbling” strategy when foraging on this task that involved fully submerging the bill and vigorously churning the water around them (Dunn’s multiple comparisons test, multiplicity-adjusted *p* < 0.05), whereas Muscovy ducks shifted to a “surface dabbling” strategy, skimming the water’s surface and producing very little agitation (Figure 2F).

## 3 Discussion

We sought to evaluate tactile foraging performance in distantly related Pekin and Muscovy ducks. Our results demonstrate that both species can successfully discriminate between edible and inedible objects and associate objects of different size and shape with reward. This suggests both species have a reasonably high level of tactile acuity in the bill when conditions demand its use. In fact, individual differences on this task explained more of the variance in the number of worms eaten than interspecies differences. However, only Pekins were able to be conditioned using inedible objects of the same size and shape but different hardness. This result bears out and extends the prior studies on wild mallards and provides a novel point of comparison in the Muscovy duck, whose tactile foraging ability has not previously been studied. That Muscovy could not perform the hardest tactile conditioning task in our study is consistent with time-activity budget analysis of Muscovy ducks spending only 1.4% of their time foraging by dabbling or probing in soil (both considered tactile foraging methods) compared to 25% foraging by grazing or gleaning from the ground, which are presumably less tactile (Downs et al., 2017).

Interestingly, during training on the hard versus soft discrimination task, both species easily learned to associate hard plastiline distractors with reward. However, during testing when the reward was absent, Muscovies spent more time foraging in the bowl containing soft plastiline cylinders, which were never paired with reward on either conditioning task. This suggests they may have been using taste cues during training, and in absence of these cues they shifted to preferring the soft cylinders because of their similarity to natural food items such as mealworms or other insect larvae. Further, Muscovies behavior during foraging also shifted on this task, albeit to a less effective strategy. Indeed, the distinct performances of Pekin and Muscovy ducks on this task could be explained in the light of both tactile acuity and instinctual tendencies, both of which could also play roles in the foraging abilities of wild ducks in their natural setting. We observed domestic Pekins using a difference in hardness as an indirect indicator of the presence of food. In the wild such an ability may aid in prey detection, where the softness of substrates or the shape and softness of aquatic vegetation could serve as an indirect indicator that the duck is foraging in an environment suitable for specific prey, like amphipods or other aquatic invertebrates. Furthermore, females learned the conditioning tasks faster than males across both species. Because these studies were performed at the onset of sexual maturity, perhaps this is indicative of many female waterfowl’s motivation to consume protein-rich insect larvae during the egg-laying period (Swanson et al., 1985). Further studies presenting distractors in a counterbalanced design across different life stages would be necessary to more fully understand the role tactile ability could play in seasonal and sex differences in foraging behavior.

Several neuroanatomical features may contribute to species differences in tactile discrimination between hard and soft objects. The mallard and Pekin duck have large PrVs (Gutierrez-Ibanez et al., 2009), and many neurons in the trigeminal ganglia express Piezo2, suggesting an expanded neural representation of tactile information from the bill. This has not been measured in the Muscovy duck; however, other members of tribe Cairinini (genus *Aix*) have small PrVs and very few mechanosensitive neurons in the trigeminal ganglia (Dubbeldam, 1990; Schneider et al., 2019). The bills of ducks contain numerous sensory corpuscles which can be found clustered in papillae in the maxillary and mandibular nails, making up a touch-sensitive region known as the bill tip organ. Filter-feeding ducks, such as the mallard, have significantly more corpuscle-containing papillae than species using other means of foraging (Avilova et al., 2018). Only a few studies exist examining Muscovy mouth morphology (Abdalla et al., 2018), and these do not quantify corpuscle density. Ducks also possess repeating comb-like ridges located along the edges of the mouth’s interior, known as lamellae, which are traditionally believed to play a role in straining food items from water. Zweers et al. (1977) described a model for the straining process in mallards, likening the mouthparts to a suction pump (Zweers et al., 1977). The piston-like movement of the tongue draws water and food particles into the rostral end of the mouth, whereby the water is expelled past the lamellae. A comparison of Pekin and Muscovy mouth morphology, including lamellae, could provide insights into how mouth morphology influences tactile ability.

Our tactile discrimination task assesses a distinct type of tactile acuity compared to previous work demonstrating Anatini could discriminate between particles of different sizes, which could be accomplished through size exclusion by lamellae. For instance, a study of mallard and shoveler ducks found that when the interlamellar space was increased by ablating a subset of lamellae filter feeding ducks performed worse on particle filtering tasks. In contrast, the dimensions of our plastiline distractors of varying hardness far exceed the interlamellar space, and are identical in size, and thus could not be differentiated by size exclusion using lamellae. Pekin ducks presumably benefit on our task from increased vibrotactile feedback consistent with a high density of Herbst and Grandry corpuscles in the bill, particularly in the bill tip organ (Berkhoudt, 1979). Ultimately, which neuroanatomical adaptations in tactile processing are most advantageous for tactile discrimination remains to be determined (Wylie et al., 2015). Since Pekin and Muscovy both have fully sequenced genomes they provide a compelling opportunity for further examination of the molecular/genetic basis of tactile acuity in waterfowl, as well as general principles of the evolution of tactile acuity.

Another intriguing area of future investigation is differences in foraging ability in domesticated and wild mallards and Muscovies. While we have demonstrated the Pekin duck as having higher tactile ability than Muscovy, mallards may have superior tactile ability to that of our meat-type Pekin ducks. Prior studies have found mallards foraging for peas among plastiline distractors in wet sand left no lamella marks on plastiline, raising the intriguing possibility that they can use remote touch, as has been observed in other water birds such as *Caladris canutus* (Piersma et al., 1998). In our studies, plastiline triangles appeared extensively chewed after testing, whereas in the hard versus soft tactile conditioning task there were few lamella markings on plastiline. Likewise, wild Muscovies may have differing abilities from domesticated Muscovies.

Though Anatini is a diverse group, the diets of many waterfowl overlap (Thomas, 1982). Our present study suggests that each species may have evolved to benefit from different feeding niches. If tactile sensitivity is better evolved in certain duck species, these species may be better suited to take advantage of the available food resources (Hitchcock et al., 2021). Characterizing tactile ability as well as the neural features that underpin behavior (e.g., pathways necessary for somatosensory development and specification, craniofacial development, and tactile response tuning of trigeminal and upstream neurons) across a wider range of species could provide a crucial lens to examine the evolution of foraging behavior. For instance, the lesser scaup (*A. affinis*), a diving duck, has the same percentage of mechanosensitive neurons in its trigeminal ganglia as the Pekin duck, but more of these neurons have rapid inactivation kinetics (Schneider et al., 2019). In contrast, the ruddy duck has relatively few mechanosensitive TG neurons but the largest PrV among waterfowl (Gutierrez-Ibanez et al., 2009). Using the behavioral methods outlined here to characterize foraging ability in other species of waterfowl, such as geese or diving ducks, with different foraging preferences or aptitudes to a standard domesticated mallard model could provide important insights into which adaptations most meaningfully contribute to the breadth of tactile abilities across species in Anseriformes.

## 4 Materials and Methods

### 4.1 Animals and General Procedure

All procedures were approved by the University of Kentucky Institutional Animal Care and Use Committee under protocol # 2021-3810. Pekin duck eggs were obtained from Maple Leaf Farms (Cromwell, IA, United States) and hatched onsite. Day-old Muscovy ducklings were shipped from Freedom Ranger Hatcheries (Reinholds, PA, United States). Pekin and Muscovy ducks were raised separately in outdoor hanging wire aviaries for the first 4 months of life, then transferred for the next 4 months to a more secure metal frame outdoor aviary (12′ × 11′ × 6.5′) with concrete floors and large flake pine shavings as bedding. Food in hanging feeders (Mazuri starter pellets, then 19% protein pellets) and water in 15-gallon poultry drinkers were available *ad libitum.* Environmental enrichment included daily enrichment feeding (peas, mealworms, grain, or greens), large tubs of water for bathing and wading, and hanging shiny objects for pecking (compact disks, aluminum cans filled with mealworms). All training and testing experiments were done within a 30′ × 44.5′ × 31′ cage placed within the aviaries.

### 4.2 Experiment #1: Increasing Ratio of Distractors to Targets

#### 4.2.1 Training

To assess tactile foraging ability, we first trained Pekin and Muscovy ducks to forage in muddy water for mealworms hidden among inedible distractors of a similar shape and size made of plastiline clay (Roma Plastilina, Chavant Inc., Macungie, PA, United States). Mealworms (Josh’s Frogs, Owasso MI, United States) were prepared a day in advance by drowning in water at 4°C to prevent floating or movement. Inedible distractors were prepared by extruding plastiline clay into 2 mm diameter × 18 mm cylinders. Muddy water was prepared from strained local mud and poured into a 4 L, 10.5 ″ diameter rubber feeding pan. As an initial training task, ducks were presented with muddy water containing 50 mealworms and 50 plastiline distractors and allowed to forage for 2 min (data not shown).

#### 4.2.2 Testing

Tests for foraging ability were conducted with the same setup as training, but feeding bowls contained 20 mealworms and differing ratios of worms to distractors in the following order: 1:25, 1:100, 1:50 (i.e., 40, 80, 60 g plastiline; 4 g = approx. 50 pieces). After each experiment, mealworms were counted and remaining plastiline was dried and weighed. A camera (Raspberry Pi 4B/HQ camera or GoPro Fusion) was placed above the feeding area and used to record each duck’s feeding behavior at 30–60 FPS. Additionally, the 1:50 test was repeated but with clear water to determine if introduction of visual information during foraging might aid the ducks in feeding. No additional training was introduced for this visual test. Testing on the 1:50 conditions coincided with the event of sexual maturity/breeding season in both species, during which many ducks did not forage at all during testing. Thus, these conditions were tested twice and in cases where ducks foraged on both days results were averaged.

### 4.3 Experiment # 2: Operant Conditioning

#### 4.3.1 Training

To rule out the use of gustatory or olfactory cues which may have been present in experiment #1, shaping behavior was used to train ducks on a two-alternative choice task of foraging between two bowls (Figure 2A). Training was done over 4 weeks, with a maximum of 3 days between training sessions (3–11 trials per task). Initially, ducks were given five mealworms in one of two bowls in randomized order in clear water (task i), then in muddy water (task ii). Worms were then placed in the container with 20 g (∼50 pieces) of extruded isosceles triangles made from soft plastiline (Prima Plastilina) (5.6 mm × 7 mm side length × 18 mm long) in one bowl, while the other bowl contained 20 g (∼120) soft plastiline cylinders (task iii). Ducks were given both bowls simultaneously. In other words, ducks learned to associate the triangles with the reward of locating worms while foraging during the training process. Ducks success on training trials (task i–iii) was quantified as the ability to actively forage for 5 s in the bowl containing the worms in under 20 s on three consecutive trials. Once ducks met this criterion on a given task they were moved on to the next task. To ensure ducks retained their learning, successful ducks were trained on Learning Task iii for at least 2 days. On the final 1–2 days of training on task iii the number of mealworms was reduced to three.

#### 4.3.2 Testing: Size and Shape Discrimination

After ducks were taken through the series of training tasks associating the food reward (mealworms) with a certain plastiline stimulus (Figure 2A), ducks were tested once to assess if they correctly learned food association with plastiline triangles. On testing day, ducks were given one rewarded trial with the same setup as task iii of training: two bowls of muddy water were placed in front of each duck, one containing mealworms and triangles, the other containing cylinders and no worms; ducks were allowed to forage for 45 s. Immediately following this rewarded trial, ducks were given one unrewarded trial with the same setup but no worms were included with the triangles (Figure 2C). Testing videos were scored by two to three independent observers at least one to two of which were blind to condition and identity of individual ducks to measure time spent foraging in each bowl (dependent variable) by each duck during the 2-min trials.

#### 4.3.3 Training and Testing: Hardness Discrimination

Ducks successful on the previous task were retrained for 3 days on a hardness discrimination task by pairing worms with hard plastiline cylinders (Roma plastiline) while the unrewarded bowl contained the same type of soft plastiline cylinders to increase similarity to the previous task. Ducks that met our criteria for success (foraging in the correct bowl in under 20 s on three consecutive trials) on this new task were then tested to determine which bowl they spent more time foraging in (dependent variable) for 2 min with no mealworms present (Figure 2D).

### 4.4 Analysis

Data were analyzed using JMP Pro, Igor Pro and Graphpad Prism. All statistical tests were either two-tailed *t*-tests (t statistic reported) or linear mixed models (F statistic reported) unless otherwise noted. If data were normally distributed (Shapiro Wilk test), we performed two-tailed *t*-tests for pairwise comparisons or ANOVA/linear mixed model for multiple comparisons. When normality assumptions were violated the Kruskal-Wallis test was used. Linear mixed models were used in lieu of ANOVA to accommodate missing values from ducks that did not forage on individual test days, with subjects [species] included as a random intercept. Independent variables were number of worms eaten, worms eaten per minute, or time spent foraging in the correct bowl (on unrewarded test conditions). Dependent variables were species, sex, and test condition. One Pekin outlier was removed from 1:100 task who foraged for only 10 s (160 worms/min) to ensure residuals were normally distributed. Training data was coded as a nominal variable and analyzed using logistic regression in JMP Pro. Figures were made using Adobe Illustrator. Error bars on graphs represent S.E.M. unless otherwise noted.

## Data Availability

The raw data supporting the conclusion of this article will be made available by the authors, without undue reservation.
